# The Vestibular Column in the Mouse: A Rhombomeric Perspective

**DOI:** 10.3389/fnana.2021.806815

**Published:** 2022-01-31

**Authors:** Carmen Diaz, Joel C. Glover

**Affiliations:** ^1^Department of Medical Sciences, School of Medicine and Institute for Research in Neurological Disabilities, University of Castilla-La Mancha, Albacete, Spain; ^2^Department of Molecular Medicine, Institute of Basic Medical Sciences, University of Oslo, Oslo, Norway

**Keywords:** hindbrain, rhombomere, vestibular nuclei, hodology, patterning

## Abstract

The vestibular column is located in the hindbrain between the sensory auditory (dorsal) and trigeminal (ventral) columns, spanning rhombomeres r1 (or r2) to r9. It contains the vestibular nuclear complex that receives sensory innervation from the labyrinthine end organs in the inner ear. Gene expression studies and experimental manipulations of developmental genes, particularly *Hox* genes and other developmental patterning genes, are providing insight into the morphological and functional organization of the vestibular nuclear complex, particularly from a segmental standpoint. Here, we will review studies of the classical vestibular nuclei and of vestibular projection neurons that innervate distinct targets in relation to individual rhombomeres and the expression of specific genes. Studies in different species have demonstrated that the vestibular complex is organized into a hodological mosaic that relates axon trajectory and target to specific hindbrain rhombomeres and intrarhombomeric domains, with a molecular underpinning in the form of transcription factor signatures, which has been highly conserved during the evolution of the vertebrate lineage.

## Introduction

**FIGURE 1:**
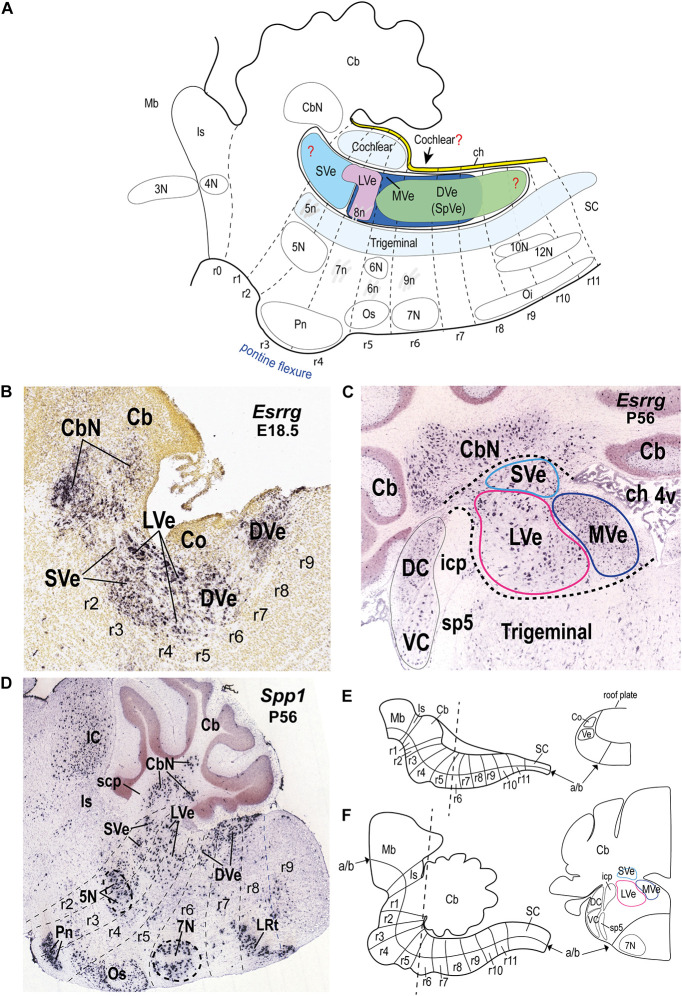
Location of the vestibular column and its main nuclear components in the hindbrain alar plate of the mouse. **(A)** Schematic sagittal representation of the rostrocaudal extension of the vestibular column in relation to the rhombomeres and neighboring cochlear and trigeminal columns. Anatomical landmarks with known rhombomeric locations are indicated. Red question marks indicate uncertain rostral and caudal limits of the vestibular column and uncertain caudal limit of the cochlear column. The trigeminal column extends up to r2 (Oury et al., 2006). Schema based on Farago et al. (2006); Oury et al. (2006); Pasqualetti et al. (2007); Tomás-Roca et al. (2016); Watson et al. (2017b); Martinez-de-la-Torre et al. (2018); García-Guillén et al. (2021). Rostral is to the left and dorsal is up. **(B)** Sagittal section of a mouse brain at E18.5 showing *Esrrg in situ* hybridization signal in identified vestibular nuclei (experiment 100071978; image 9 of 16: Allen Developing Mouse Brain Atlas). **(C)** *Esrrg* expression in a coronal section through the vestibular column at P56 (experiment 73616033; image 88 of 109: Allen Developing Mouse Brain Atlas). Medial is to the right. Black dashed lines indicate the contours of the indicated classical vestibular nuclei. Note the presence of *Esrrg*-expressing cells in the superior, lateral and medial vestibular nuclei (LVe, MVe, and SVe), and cerebellar and cochlear nuclei (CbN, DC, and VC). **(D)** Expression of *Spp1* in the hindbrain at P56 (experiment 513488; image 11 of 20: Allen Developing Mouse Brain Atlas). Rhombomeric boundaries are delineated based on the Reference Atlas of the Allen Developing Mouse Brain Atlas. **(E)** (Left) Schematic sagittal view of the hindbrain from the isthmic rhombomere to rhombomere 11 at approximately E10.5 [modified from Puelles et al. (2013); their Figure 1C]. (Right) Schematic transverse section at the r5-r6 level indicated in the left panel [adapted from Di Bonito and Studer (2017); their Figure 5B]. **(F)** (Left) Schematic sagittal view of the hindbrain from the isthmic rhombomere (r0) to r11 at a postnatal stage [modified from Puelles et al. (2013); their Figure 1E]. (Right) Schematic transverse section at the r5-r6 level indicated in the left panel [modified from the mouse brain atlas of Paxinos and Franklin (2013); their Figure 80]; a similar level is photographed in panel **(C)**. 3N, oculomotor nucleus; 4N, trochlear nucleus; 4v, fourth ventricle; 5N, motor trigeminal nucleus; 5n, motor trigeminal nerve; 6N, abducens nucleus; 6n, abducens nucleus; 7N, facial nucleus; 7n, facial nerve; 8n, vestibulo-cochlear nerve; 9n, glossopharyngeal nerve; 10N, dorsal vagal motor nucleus; 12N, hypoglossal motor nucleus; a/b, alar/basal boundary; Cb, cerebellum; CbN, cerebellar nuclei; ch, choroid tela; Co, cochlear column; DC, dorsal cochlear nucleus; DVe (SpVe), descending (spinal) vestibular nucleus; IC, inferior colliculus; icp, inferior cerebellar peduncle; Is, isthmus; LRt, lateral reticular nucleus; LVe, lateral vestibular nucleus; Mb, midbrain; MVe, medial vestibular nucleus; Oi, inferior olive; Os, superior olive; Pn, pontine nuclei; r, rhombomere; r, rhombomere; SC, spinal cord; scp, superior cerebellar peduncle; sp5, spinal trigeminal tract; SVe, superior vestibular nucleus; VC, ventral cochlear nucleus; Ve, vestibular column.

In vertebrates, the sensorimotor vestibular system receives information related to the position and movement of the head with respect to the gravitational field and utilizes this to control body posture and stabilize gaze. Although the structure and organization of the sensory apparatus varies among vertebrate classes, this principle function remains the same. To take the mammalian situation as an example, angular acceleration of the head is detected by the three orthogonal semicircular canals, whereas linear acceleration and static position of the head are detected by the two otolith organs, the utricle and the saccule (Fritzsch and Beisel, 2001, 2004; Goldberg et al., 2012). The information from these peripheral sensory organs in the inner ear is channeled by the sensory afferents in the vestibular nerve to the vestibular nuclei in the hindbrain. The vestibular afferent fibers, which are separate from the auditory fibers (Goldberg et al., 2012; their Figure 6.8), enter the hindbrain through the alar plate of rhombomere r4 (Figure 1A). From there they bifurcate into longitudinal descending and ascending branches to innervate neurons in the superior, lateral, descending (spinal or inferior) and medial vestibular nuclei within the vestibular column (Sato et al., 1989; Büttner-Ennever, 2000; Goldberg et al., 2012, their Figure 6.9; Díaz and Puelles, 2019, their Figure 1). The vestibular nuclei integrate the vestibular information together with signals from the spinal cord, the cerebellum, and the visual system. Vestibular projection neurons within these nuclei transmit the processed information to specific premotor and subcortical motor centers (ocular, reticular, and spinal) to control the extraocular and body muscles that effect rapid adjustments in eye position, body posture and movement. The vestibular nuclei have substantial reciprocal connections with the cerebellum that modulate the motor pathways controlling balance and gaze and compare intention and action to adjust the signals transmitted to the motor centers. Vestibular information also reaches cortical areas through thalamic nuclei, contributing to a subjective perception of the external environment as it relates to movement. The vestibular nuclei are abundantly interconnected through bilateral vestibulo-vestibular and vestibulo-reticulo-vestibular connections [see previous reviews in Büttner-Ennever (2000); Highstein and Holstein (2006), Goldberg et al. (2012)].

## The Vestibular Column in the Hindbrain

The hindbrain is subdivided dorsoventrally into four histogenetically defined longitudinal plates (roof, alar, basal, and floor). Expression of the *Pax3/7* genes characterize the alar plate (Manuel and Price, 2005) whereas the basal plate expresses *Nkx2.1* and *Nkx6.1* (Puelles et al., 2004). The roof plate gives rise mainly to the choroidal tela, the alar plate to somatosensory and viscerosensory columns, and the basal plate to the somatomotor and visceromotor nuclei. The derivative of the floor plate is a palisade of radial astroglial named the median raphe (Puelles, 2013).

**FIGURE 2:**
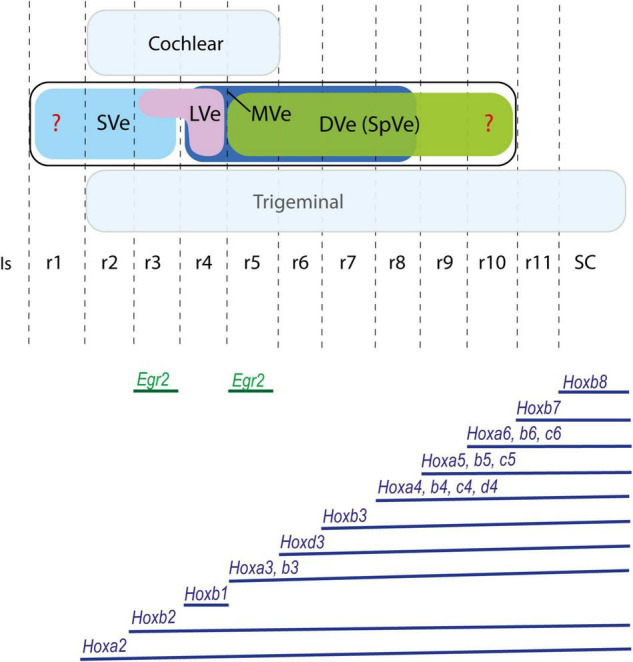
Proposed rostrocaudal plurisegmental origins of the cytoarchitectonically defined vestibular nuclei **(upper panel)** and associated *Hox* expression pattern **(lower panel)** in the mouse. *Egr2* gene, expressed selectively in rhombomeres 3 and 5, is also illustrated. Dorsal cochlear and ventral trigeminal columns are highlighted in light blue. Red question marks indicate uncertain rostral and caudal limits of the vestibular column. The *Hox* expression pattern shows species variability and differences throughout development. Schemes based on Pasqualetti et al. (2007); Chen et al. (2012); Di Bonito et al. (2015); Tomás-Roca et al. (2016); Krumlauf and Wilkinson (2021). DVe (SpVe), descending (inferior) vestibular nucleus; Is, isthmus; LVe, lateral vestibular nucleus; MVe, medial vestibular nucleus; r, rhombomere; SC, spinal cord; SVe, superior vestibular nucleus.

The vestibular column is located within the alar plate, intercalated between the dorsalmost sensory acoustic (or cochlear) column, which is rostrocaudally less extensive, and the ventralmost and more extensive trigeminal descending column [review in Puelles (2013); Figure 1A]. The vestibular column is bounded rostrally by the isthmocerebellar region that contains, among other entities, the cerebellum, the noradrenergic locus coeruleus and the parabrachial complex (Aroca and Puelles, 2005; Watson, 2010; Watson et al., 2017b; Figure 1A). At its caudal pole, the vestibular column contacts the choroidal tela dorsally (due to the more restricted caudal extent of the cochlear column), and the dorsal column nuclei caudally (Farago et al., 2006; Tomás-Roca et al., 2016; Figures 1A, 2).

The locations of vestibular progenitor subdomains along the dorsoventral axis is a consequence of the competition between dorsalizing diffusible signals secreted from the ectoderm and roof plate [bone morphogenic proteins (BMPs) and wingless and int-1 (WNTs)] and ventralizing signals from the notochord and floor plate [sonic hedgehog (SHH); Placzek et al., 1991; Ericson et al., 1995; Ulloa and Martí, 2010; Placzek and Briscoe, 2018]. These signaling gradients activate the expression of distinct transcription factors that contribute to specify molecularly the dorsoventral longitudinal columns in eight alar and six basal progenitor microzones [Takahashi and Osumi, 2002; Melton et al., 2004; Sieber et al., 2007; Gray, 2008; Storm et al., 2009; reviewed in Puelles (2013); see his Figure 10.4b; Di Bonito and Studer, 2017; their Figure 2]. Tangential migrations among these dorsoventral longitudinal subdomains also occurs, contributing to an increase in the complexity of the hindbrain [reviewed in Puelles (2013); Di Bonito and Studer (2017)]. Although their dorsoventral origins have not been fully elucidated, it is clear that vestibular neurons arise from multiple dorsoventral progenitor domains, including from outside the alar plate (see Lipovsek and Wingate, 2018; Lunde et al., 2019). Tangential migrations from these diverse dorsoventral origins converge into the vestibular column, which must thereafter be imbued with a specific molecular profile that attracts the vestibular afferents from the vestibular ganglion in contrast to the acoustic column that attracts the auditory afferents from the spiral ganglion (Fritzsch and Beisel, 2001, 2004; Glover, 2020b). Both populations of sensory afferents arrive together but segregated within the eighth nerve, entering through a gateway located at the r4 alar plate, close to the lateral vestibular nucleus (8n, Figure 1A; see also Díaz and Puelles, 2019).

Expression of the genes *Spp1* and *Esrrg* delimit quite well the vestibular column from the neighboring cochlear and trigeminal columns (Figures 1B–D); both markers have been correlated previously with the vestibular column (Lee et al., 2001; Lunde et al., 2019). At E18.5 and younger stages, *Esrrg* is expressed mainly in cells of the vestibular column, and in the rostralmost cerebellar nuclei (Figure 1B; Lunde et al., 2019; ©2008 Allen Developing Mouse Brain Atlas^1^). However, scattered *Esrrg-*positive cells are also present in the cochlear and trigeminal nuclei at postnatal stages (Figure 1C). *Esrrg* encodes the estrogen-related receptor gamma, which is also expressed in hindbrain cells of zebrafish and amphioxus (Bardet et al., 2005). *Spp1* expression also demarcates the vestibular column although it has a broader expression pattern than *Esrrg* at postnatal stages (P14 onward), especially including basal cell populations such as the trigeminal and facial cranial motor nuclei, and pontine, superior olivary and lateral reticular nuclei (Figure 1D). Nevertheless, at E18.5 and P4, *Spp1* expression is mainly restricted to the vestibular column (possibly in the lateral vestibular nucleus and rostral part of the descending nucleus) and the cerebellar nuclei (©2008 Allen Developing Mouse Brain Atlas), and its expression pattern is comparable in the developing rat hindbrain (Lee et al., 2001). *Spp1* encodes a secreted phosphoprotein named SPP1 or osteopontin, which has been related to developmental processes such as axon myelination (Selvaraju et al., 2004; Jiang et al., 2019), proliferation, differentiation, and migration (Kalluri and Dempsey, 2012).

Along the anteroposterior axis, the vestibular column in the mouse extends roughly from rhombomere r1 to r9, although its rostral and caudal limits are not yet clearly established (Figure 1A). Thus, the rostral extension of the vestibular column to r1, based on quail-chicken grafts at stages HH10-11 (Marín and Puelles, 1995), may require further experimental analysis, since the prospective r1/r2 boundary in the chicken embryo was assumed by these authors, without using molecular markers, as lying approximately at the middle of pro-rhombomere A of Vaage (1969). *Hoxa2* expression delimits the r1/r2 boundary at stages HH11-12 (Aroca and Puelles, 2005; Aroca et al., 2006) and could be used as a better landmark for assessing the rostral limit of the vestibular column in future studies of the chicken embryo. Some evidence suggests that the vestibular column begins at r2, in parallel with the cochlear column (Farago et al., 2006), without any component in r1. This stems from the presence of a molecularly distinct isthmocerebellar (prepontine) region derived from r0-r1 (Watson, 2010; Watson et al., 2017a,Watson et al., 2019) that contains non-vestibular cell groups. For instance, the strongly AZIN2-labeled trigeminal mesencephalic nucleus, which is thought to extend from r1 rostrally, lies immediately rostral to the moderately AZIN2-labeled vestibular column in the mouse (Martinez-de-la-Torre et al., 2018; their Figure 1C). With respect to the caudal pole, the vestibular column apparently ends in r9 in mouse, contacting the dorsal column nuclei (Tomás-Roca et al., 2016), although it has been proposed to extend at least to r10 in the chicken (Cambronero and Puelles, 2000).

No experimental data exist on the rhombomeric origins of the classical vestibular nuclei in mouse. However, Pasqualetti et al. (2007) proposed a tentative correlation between their rhombomeric map of the mouse vestibular projection neurons with classical cytoarchitectonic vestibular nuclei from r2 to r5. Based on their proposals, we can predict that the superior vestibular nucleus derives at least from r2, the lateral vestibular nucleus mainly from r4 and the descending and medial vestibular nuclei have components derived at least from r5 and r6. Fate mapping studies in chicken suggest that the superior vestibular nucleus derives from r1 and r2, the lateral vestibular nucleus from r4 with a small component in r3, the descending (or inferior) vestibular nucleus from r5-r10, and the medial vestibular nucleus from r4-r8 (Marín and Puelles, 1995; Cambronero and Puelles, 2000; Figures 1A, 2).

During the development of the hindbrain, in parallel with the increased curvature of the longitudinal axis at the pontine flexure (almost 90 degrees in mouse; Watson et al., 2017a,b; Figure 1A), morphological changes occur in the transverse plane of the hindbrain that affect the mature anatomical positions of the vestibular nuclei, and their relationships to neighboring structures such as the acoustic/cochlear nuclei (topologically dorsal) and the cerebellum (topologically rostral). In particular, the dorsoventral axis becomes pitched laterally as the roof plate expands to form the roof of the fourth ventricle (future choroidal tela), and thus obtains a more mediolateral orientation in the mature state. Because of the morphological transformations of the rostrocaudal and dorsoventral axes, “transverse” sections through the vestibular nuclear complex in mature hindbrains typically do not provide a clear indication of the original axes, thus complicating the interpretation of anatomical findings as they relate to rhombomeric organization and dorsoventral patterning (see Figures 1E,F). For instance, in the transverse section illustrated in Figure 1C, the cochlear nuclei, which are topologically dorsal to the vestibular column, appear to lie lateral to the vestibular nuclei. Moreover, the cerebellum, a complex structure derived from the isthmic (r0) and r1 rhombomeres, rostral to the vestibular column, enlarges greatly and eventually lies dorsal to the vestibular column (Aroca and Puelles, 2005; Figures 1A,C–F).

## Molecular Profiling of the Classical Vestibular Nuclei

Different anteroposterior identities are provided to each rhombomere by virtue of the combinations of *Hox* genes expressed within each segment (Trainor and Krumlauf, 2000; Marín et al., 2008; Tümpel et al., 2009; Tomás-Roca et al., 2016; Krumlauf and Wilkinson, 2021; Figure 2). Therefore, at least eight rostrocaudal vestibular subpopulations, spanning eight consecutive rhombomeres (r2-r9) can be conceived on the basis of differential *Hox* gene expression alone. However, classically only four main vestibular nuclei have been recognized based on cytoarchitectonic characteristics: the superior, lateral, descending (inferior or spinal), and medial nuclei, with some additional minor cell groups such as cell group Y and the interstitial nucleus of the vestibular nerve also discernible (Brodal and Pompeiano, 1957; Brodal, 1974; Wold, 1976; Büttner-Ennever, 2000). These vestibular nuclei are plurisegmental in origin since all relate to at least two rhombomeres as fate-mapped in chicken (Marín and Puelles, 1995; Cambronero and Puelles, 2000; Díaz et al., 2003; Marín et al., 2008; Figures 1A, 2), apart from the lateral vestibular nucleus, which relates mainly to r4, at least in the chicken (Marín and Puelles, 1995; Díaz et al., 2003). In the mouse, a segmental analysis of medulla oblongata correlated the expression pattern of *Hox* genes (groups 3–8) with the vestibular column and other longitudinal structures (Tomás-Roca et al., 2016). Their results suggest a plurisegmental subdivision of the vestibular column from r7 to r11.

**FIGURE 3:**
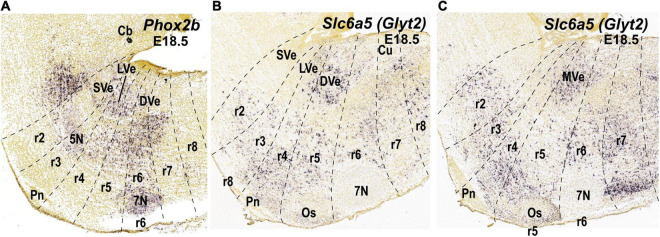
Expression of selected molecular markers in the cytoarchitectonic vestibular nuclei of the mouse according to existing literature in mammals (see main text). Sagittal sections were obtained from ISH data of the Allen Brain platform. Anatomical landmarks with known rhombomeric locations are indicated, and rhombomeres are delimited based on Reference Atlas (Allen Developing Mouse Brain Atlas). **(A)** *Phox2b* expression (experiment 100085299; image 7 of 18); **(B,C)** *Slc6a5* expression (experiment 100057240; images 10 and 11 of 19, respectively). 5N, motor trigeminal nucleus; 7N, facial nucleus; Cb, cerebellum; CbN, cerebellar nuclei; Co, cochlear column; Cu, cuneate nucleus; DVe, descending vestibular nucleus; LVe, lateral vestibular nucleus; LVe (d), lateral vestibular nucleus (dorsal Deiterśnucleus); LVe (v), lateral vestibular nucleus, ventral part; MVe, medial vestibular nucleus; Os, superior olive; Pn, pontine nuclei; r, rhombomere; SVe, superior vestibular nucleus.

**FIGURE 4:**
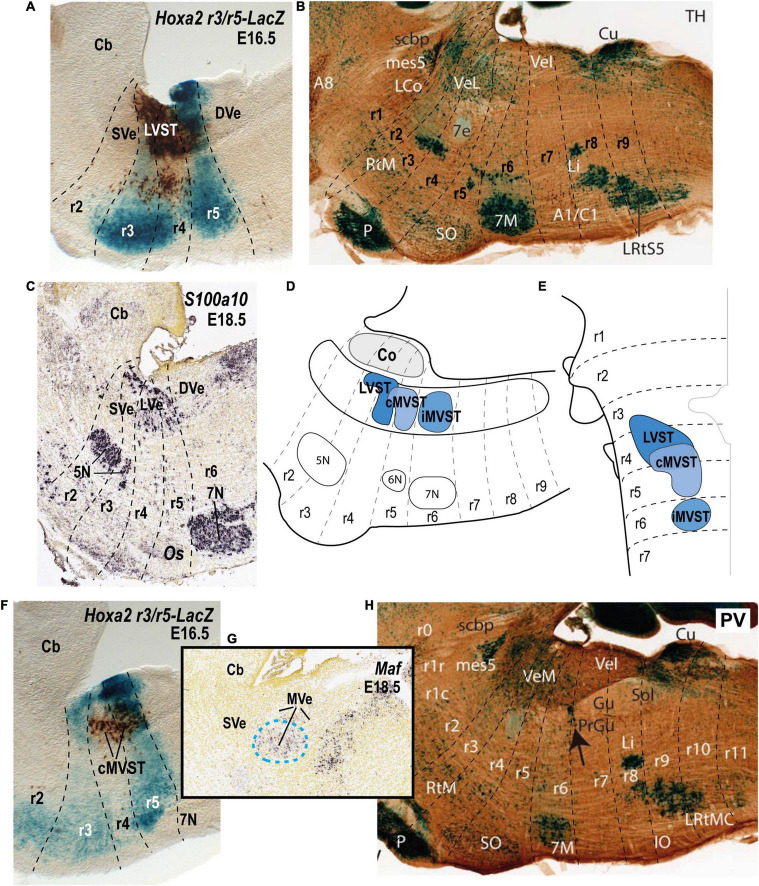
Relationship of the vestibulospinal neuron groups to the rhombomeres in the mouse. **(A)** Lateral sagittal section illustrating retrograde biotin-conjugated dextran amine (BDA) labeling of the lateral vestibulospinal group (LVST) relative to the r3 and r5 domains in a r3/r5:lacZ transgenic mouse at E16.5 (see Pasqualetti et al., 2007). **(B)** Sagittal section of an adult AZIN1-lacZ mouse counterstained with antibodies to tyrosine hydroxylase (TH). Note the weak lacZ signal in the lateral vestibular nucleus (VeL), and the stronger lacZ signal in the mesencephalic trigeminal nucleus (mes5). Other recognizable characteristic landmarks are labeled [modified from Martinez-de-la-Torre et al. (2018); their Figure 1C]. **(C)** *S100a10* expression predominantly in the lateral vestibular nucleus (LVe; blue dashed line) in a sagittal section downloaded from the Allen Developing Mouse Brain Atlas (experiment 100055828; image 9 of 18). **(D,E)** Schematic comparison of the vestibulospinal neuron groups according to fate map reported by Pasqualetti et al. (2007). **(F)** Medial sagittal section showing retrograde BDA labeling of the contralateral medial vestibulospinal tract group (cMVST) in the r3/r5:lacZ transgenic mouse at E16.5 (Pasqualetti et al., 2007). **(G)** Expression of *Maf* at E18.5 (experiment 100132196; image 10 of 20: Allen Developing Mouse Brain Atlas). Note that *Maf*-positive cells in the rostralmost part of the medial vestibular nucleus (MVe, blue dashed line), have a location identical to that of the cMVST (compare to **F)**. **(H)** Sagittal section of an adult AZIN1-lacZ mouse counterstained with antibodies to parvalbumin (PV) showing lacZ expression in the medial vestibular nucleus (VeM). By contrast, the inferior (descending) vestibular nucleus (VeI) lacks lacZ signal [modified from Martinez-de-la-Torre et al. (2018); their Figure 2A]. 5N, motor trigeminal nucleus; 6N, abducens nucleus;7N/7M, facial nucleus; Cb, cerebellum; Co, cochlear column; DVe/VeL, descending (inferior) vestibular nucleus; LVe/VeL, lateral vestibular nucleus; MVe/VeM, medial vestibular nucleus; Os/SO, superior olive; P, basilar pontine nuclei; r, rhombomere; SVe, superior vestibular nucleus.

The lateral vestibular nucleus (LVe) has a particular profile among the vestibular nuclei, perhaps due to its main origin in r4 where *Hoxb1* is distinctly expressed. For instance, the LVe expresses *Phox2b* at early embryonic stages (Chen et al., 2012; Lunde et al., 2019; Figure 3A) and *S100a10* (Milosevic et al., 2017, Figure 4C) at late embryonic or adult stages. LVST axons, which derive primarily (but not exclusively, see Díaz et al., 2003) from the LVe, are immunoreactive for the vesicular glutamate transporter 2, VGlut2 (Du Beau et al., 2012), indicating their excitatory function (Goldberg et al., 2012). The LVST axons synapse on spinal MNs and interneurons ipsilaterally to control limb and axial musculature (Wilson and Yoshida, 1969; Grillner et al., 1970; Shinoda et al., 1986; Kasumacic et al., 2010). The LVe nucleus is subdivided into a dorsal magnocellular portion (dorsal Deiters’ nucleus) and a ventral portion containing small and medium-sized neurons (ventral Deiters’ nucleus; Brodal and Pompeiano, 1957; Brodal, 1984). Both portions contribute to the LVST, and dorsal Deiters’ also contributes to the cMVST (Lunde et al., 2019). By contrast, the rostralmost superior vestibular nucleus (SVe), mainly containing medium-sized to small cells, does not express *Phox2b* (Figure 3A) or *S100a10* (Figure 4C). Axons from SVe project rostrally and include inhibitory inputs to the oculomotor complex (Büttner-Ennever, 2000; Glover, 2003). The descending (or inferior) and medial vestibular nuclei (DVe and MVe), which each project ascending and descending axons, also do not express *Phox2b* or *S100a10* (Figures 3A, 4C), and contain neurons that express the glycine vesicular transporter *Slc6a5* and are therefore presumed to be inhibitory (Figures 3B,C) as previously shown in the rat (Tanaka and Ezure, 2004). Interestingly, *Maf*, a transcription factor-encoding gene, is expressed in the E18.5 mouse in a subpopulation of the MVe that corresponds to the cMVST group, which projects contralaterally to the rostral spinal cord (see below; Lambert et al., 2016; Lunde et al., 2019; Figure 4G). *Maf*-positive cells localize in the moderately AZIN2-lacZ expressing MVe/VeM (Martinez-de-la-Torre et al., 2018; Figure 4H).

## The Relationship of Hodologically Defined Vestibular Neuron Groups to the Rhombomeres

The connectivity of the classical vestibular nuclei offers no easily comprehensible relationship between anatomy and function. None of the nuclei exhibits a distinct function, since they all project to multiple targets, encompassing vestibulospinal, vestibulo-reticular, vestibulo-ocular, and vestibulo-vestibular projections, and have heterogeneous functional attributes, such as excitatory vs. inhibitory neurotransmitter phenotype and responses to specific patterns of afferent input (Brodal, 1974; Büttner-Ennever, 1992, 2000; Peusner et al., 1998; Barmack, 2003; Glover, 2020a,b). An alternative classification of vestibular projection neurons, obtained by combining retrograde axonal tracing and developmental fate mapping in mouse and chicken embryos, has defined vestibular neuron groups hodologically, according to their axonal pathways (ipsi- or contralateral, ascending or descending) and synaptic targets (spinal cord, oculomotor complex, or cerebellum; Glover and Petursdottir, 1988; Pétursdóttir, 1990; Glover, 1994; Díaz et al., 1998, 2003; Díaz and Puelles, 2003; Pasqualetti et al., 2007; see recent reviews in Díaz and Puelles, 2019; Glover, 2020a,b). Although the picture is not complete, these studies show that the vestibular projection neurons are organized into coherent and largely segregated groups encompassing three main vestibulospinal groups, at least four vestibulo-ocular groups, and four vestibulo-cerebellar groups, each of which can be correlated with specific rhombomeric domains (chicken: Díaz et al., 1998, 2003; Díaz and Puelles, 2003; Glover, 2003; mouse: Pasqualetti et al., 2007; Glover, 2020a,b). These hodologically defined neuron groups do not correlate strictly to the classical cytoarchitectonic nuclei, as shown in the chicken embryo (Díaz et al., 2003), indicating that hodology and cytoarchitectonics represent different types of organization, the former being more directly related to function. In the mouse, only the vestibulospinal and vestibulo-ocular neuron groups have been mapped segmentally (Pasqualetti et al., 2007; Di Bonito et al., 2015), whereas both these and the vestibulo-cerebellar neuron groups have been mapped in chicken (Díaz et al., 1998, 2003; Díaz and Puelles, 2003). Other types of vestibular projection neurons (vestibulo-vestibular, vestibulo-thalamic) remain to be characterized according to rhombomeric organization. Most of the analyzed vestibular projection neuron groups are plurisegmental in origin with some exceptions, such as the lateral vestibulospinal group (LVST group) and the ipsilateral medial vestibulospinal group (iMVST group) which derive, respectively, from r4 and r6 in the mouse and chicken (see below).

The vestibular “hodological mosaic” has been corroborated both in amniotes and anamniotes [see below; reviewed in Glover (2000; 2003, 2020a,b); Díaz and Glover (2002); Di Bonito et al. (2013); Straka and Baker (2013); Díaz and Puelles (2019)]. This implies that the patterning of the hodological mosaic reflects an evolutionarily conserved mechanism that links position, axon trajectory, and synaptic connectivity. Thus, gene expression differences between rhombomeric domains contribute to creating functional diversity among vestibular projection neuron subpopulations.

### The Vestibulospinal Neuron Groups

In the mouse embryo, as in the chicken embryo (Glover and Petursdottir, 1988; Díaz et al., 1998), three distinct and largely segregated vestibulospinal neuron groups can be distinguished (Pasqualetti et al., 2007; Figure 4). The LVST group projects ipsilaterally in the lateral vestibulospinal tract, and the iMVST and cMVST groups project, respectively, ipsilaterally and contralaterally in the medial vestibulospinal tract. The LVST group derives exclusively from r4, the cMVST group derives from r4 and r5, and the iMVST group derives exclusively from r6 (Pasqualetti et al., 2007; Di Bonito et al., 2015; Lunde et al., 2019; Figure 4). A fourth vestibulospinal projection, originating from the caudal descending nucleus (Peterson and Coulter, 1977; Peterson et al., 1978; Donevan et al., 1992a,b), has not yet been characterized developmentally.

In the mouse, the LVST group not only originates from r4 (see Auclair et al., 1999; Di Bonito et al., 2015), but also depends on the expression of *Hoxb1*, which controls r4 identity (Chen et al., 2012; Di Bonito et al., 2015). In *Hoxb1* knockout mice, the LVST neurons, along with other r4-derived neurons, including the r4-derived portion of the cMVST, are absent (Chen et al., 2012; Di Bonito et al., 2015). Some LVST neurons migrate from r4 into r3 (Pasqualetti et al., 2007; Di Bonito et al., 2015; Figures 4A,D,E). The LVST group is located largely (but not exclusively) within the r4-derived part of the LVe, intercalated between the superior and the descending (or inferior) nuclei (compare sections A and B in Figure 4). As we noted above, several molecular markers are expressed in the LVe, such as *S100a10* (Figure 4C), *Phox2b* (Figure 3A), and AZIN2 (Martinez-de-la-Torre et al., 2018; VeL in Figure 4B). The cytoarchitectonically defined LVe was previously characterized by the combined expression of the transcription factors Phox2a, Phox2b, and Lbx1 (Schubert et al., 2001; Chen et al., 2012), whereas the hodological LVST neuron group is defined by the combined expression of Phox2b, Lbx1, Esrrg, and Maf in mouse and chicken (Lunde et al., 2019; Table 1; *Esrrg*-mRNA expression in the LVe is shown in Figures 1C,D). The transcription factors Pouf3f1, Onecut1, 2, and 3 are also expressed in subpopulations within the LVST (Lunde et al., 2019; Table 1).

**TABLE 1:** Transcription factor signatures of the vestibulospinal neuron groups relative to the rhombomeres [based on Lunde et al. (2019)].

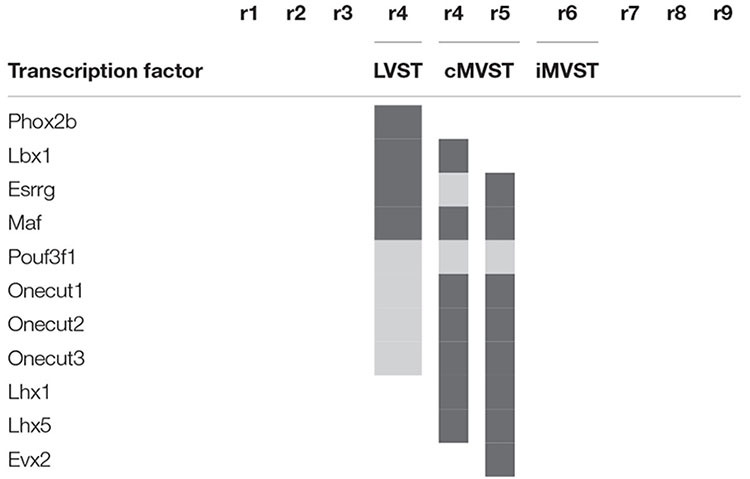

*Gray color indicates transcription factors expressed in specified vestibulospinal neuron groups.*

The cMVST group originates primarily from r5 but includes a magnocellular component that originates from r4, whereas the much smaller iMVST group originates from r6 (Pasqualetti et al., 2007; Di Bonito et al., 2015; Lunde et al., 2019; Figures 4D–F). In chicken, cMVST neurons are located primarily in the r4- and r5-derived portions of the LVe and DVe (with small proportions of neurons located in the SVe and MVe) whereas iMVST neurons are located predominantly in the r6-derived portion of the DVe (Díaz et al., 2003). cMVST neurons are defined by the combined expression of Lhx1/5, Esrrg and Maf/*Maf* (Figures 4F,G), together with Lbx1 in the r4-derived portion and Evx2 in the r5-derived portion (Lunde et al., 2019; Table 1). The r4-derived portion of the cMVST group thus shares with the LVST group its rhombomeric origin and the expression of Lbx1, and partially overlaps the LVST spatially (within the LVe), whereas the r5-derived portion of the cMVST group is distinctly Evx2-positive (Lunde et al., 2019; Table 1). These specific transcription factor signatures are conserved between mouse and chicken, indicating a shared evolutionary origin at least 300 million years into the vertebrate past (Lunde et al., 2019). These group-specific transcription factor signatures represent key candidates for regulating the differentiation, axonal navigation and synaptogenesis that specify the functional identities of the vestibular projection groups (Glover, 2003; Nieuwenhuys and Puelles, 2016; Lunde et al., 2019).

### The Vestibulo-Ocular Groups

**FIGURE 5:**
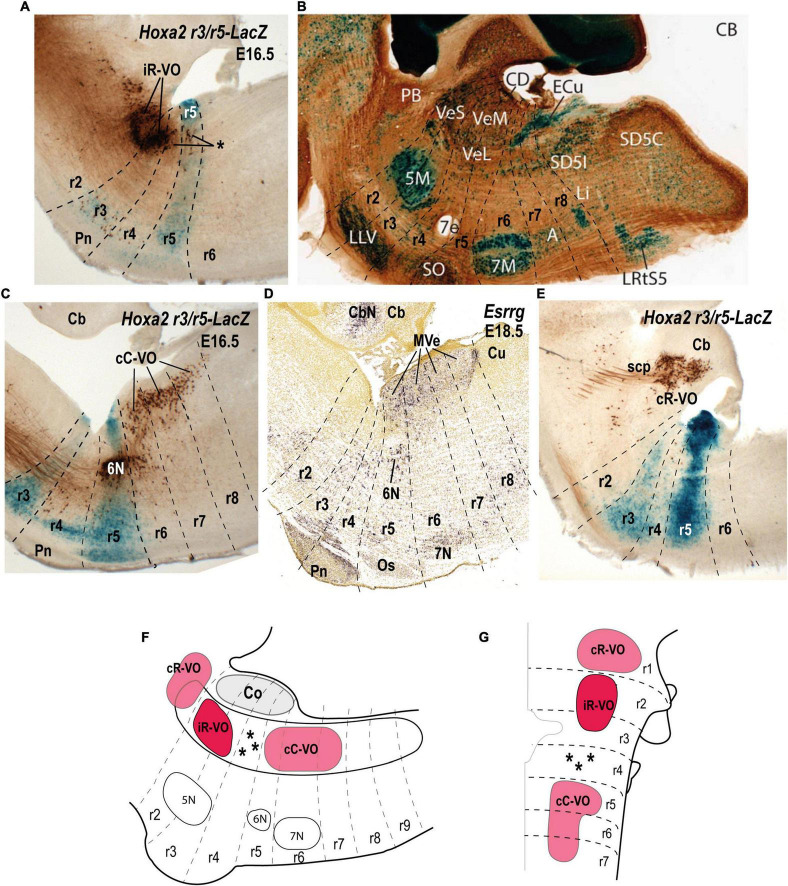
Relationship of the vestibulo-ocular neuron groups to the rhombomeres in sagittal sections of E16.5/E18.5 mouse embryos. **(A)** Biotin-conjugated dextran amine (BDA) labeling of the ipsilateral rostral vestibulo-ocular group (iR-VO) relative to the r3 and r5 domains in a r3/r5:lacZ transgenic mouse at E16.5 (see Pasqualetti et al., 2007). Asterisk indicates scattered labeled cells in r4 and r5. **(B)** Sagittal section of an adult AZIN1-lacZ mouse counterstained with antibodies to calbindin (CB). Neurons of the superior, lateral and medial nuclei are moderately AZIN2 positive [modified from Martinez-de-la-Torre et al. (2018); their Figure 1B]. **(C)** The contralateral caudal vestibulo-ocular neuron group (cC-VO) labeled by BDA in a sagittal section of a r3/r5:lacZ transgenic mouse at E16.5 (see Pasqualetti et al., 2007). This section is medial to the section shown in panel **(A)**. **(D)** *Esrrg* expression in the medial vestibular nucleus (MVe) in a sagittal section at E18.5 (experiment 100071978; image 16 of 16: Allen Developing Mouse Brain Atlas). **(E)** The contralateral rostral vestibulo-ocular group (cR-VO) labeled by BDA in a section of the r3/r5:lacZ transgenic mouse at E16.5 [from Pasqualetti et al. (2007)]. **(F,G)** Schematic comparison of the vestibulo-ocular neuron groups according to the fate map reported by Pasqualetti et al. (2007). 5N/5M, motor trigeminal nucleus; 6N, abducens nucleus; 7e, efferent facial fibers; 7N/7M, facial nucleus; Cb, cerebellum; CbN, cerebellar nuclei; CD, dorsal cochlear nucleus; ECu, external cuneate nucleus; LVe/VeL, lateral vestibular nucleus MVe/VeM, medial vestibular nucleus; Os/SO, superior olive; Pn, pontine nuclei; r, rhombomere; scp, superior cerebellar peduncle; Sol, solitary nucleus; SVe/VeS, superior vestibular nucleus.

Three vestibular neuron groups project to the rostral oculomotor complex (oculomotor and trochlear nuclei) in both chicken and mouse: two rostral groups and one caudal group, with either ipsilateral or contralateral axon trajectories (iR-VO, cR-VO, and cC-VO, Pétursdóttir, 1990; Glover and Petursdottir, 1991; Jansen, 1991; Glover, 2003; Pasqualetti et al., 2007; Figure 5). A fourth group that projects to the rostral oculomotor complex, located caudally and with an ipsilateral axon projection (iC-VO), is seen distinctly in the chicken embryo (Pétursdóttir, 1990) and amphibians (Straka et al., 2001, 2002; see below), but only at early stages in the mouse embryo (Pasqualetti et al., 2007). Vestibulo-ocular projections to the abducens nucleus have not yet been defined in the same hodological context.

With respect to the relationship between the hodologically defined vestibulo-ocular groups and rhombomeric domains, the cR-VO group relates to a single rhombomere (r1), whereas the iR-VO and cC-VO groups derive from multiple rhombomeres (r2-r3 and r5-r7, respectively; Pasqualetti et al., 2007).

The iR-VO group relates to r2 with scattered neurons in r3, an origin consistent with its location in the center of the SVe (Figures 5A,B,F,G; Glover, 2000, 2003; Díaz and Glover, 2002; Díaz et al., 2003; Pasqualetti et al., 2007). Combination of retrograde labeling with immunohistochemistry for the inhibitory transmitter GABA shows that the iR-VO group is GABA-immunoreactive (Glover, 1994), which fits neatly with the inhibitory role this group plays in vertical vestibulo-ocular reflex pathways (Jansen, 1991; Glover, 2003).

The cC-VO group is located caudal to r4 and apparently spans from r5 to r7, although its precise caudal extent has not yet been determined (Pasqualetti et al., 2007; see their Figures 5B,C; Figures 5C,D,F,G). cC-VO neurons extend lateromedially from the DVe to the MVe and further to the abducens nucleus (6N; Figure 5C). Many neurons of the MVe, where the cC-VO is mainly located, express inhibitory markers such as *Slc6a5* (Figure 3C), which encodes a glycine vesicular transporter protein (GLYT2; Tanaka and Ezure, 2004). However, the contralateral vestibulo-ocular projections are mainly excitatory (Precht, 1979), so the cC-VO group should contain predominantly excitatory neurons.

The cR-VO group is partially located in the ventral part of the caudal cerebellar peduncle in r1 (Figure 5E). This hodological cR-VO subpopulation was correlated with the infracerebellar nucleus by Pasqualetti et al. (2007), based on a comparison with mammalian literature. Perhaps this r1-related cR-VO subpopulation, whose axons project in the brachium conjuctivum, is actually patterned as part of the cerebellar anlage by the isthmic organizer, rather than as part of the vestibular column *per se*.

**FIGURE 6:**
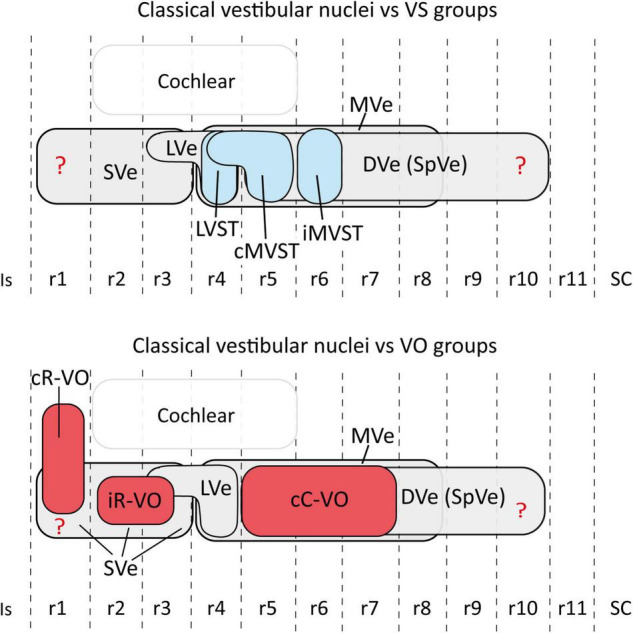
Schematic representation of the relationship between the hodologically defined vestibular neuron groups [**(upper panel)** vestibulospinal groups in blue; **(lower panel)** vestibulo-ocular groups in red] and the classical cytoarchitectonic nuclei (in gray) in the mouse. The view is from the lateral aspect. Interhombomeric limits are indicated with dash lines. Rostral is to the left and dorsal is up. Note that, despite the appearance that some groups overlap a lot (especially cC-VO with cMVST and iMVST), in 3D the overlap is minimal (aside from specific examples of overlap described in the text). The rhombomeric origins of the vestibulospinal and vestibulo-ocular neuron groups are based on Pasqualetti et al. (2007); Di Bonito et al. (2015); Lunde et al. (2019). Correlation between the rhombomeric maps of vestibular projection neurons and classical cytoarchitectonic vestibular nuclei is tentative [see proposals in Pasqualetti et al. (2007)]. cC-VO, contralateral vestibulo-ocular group; cMVST, contralateral medial vestibulospinal group; DVe (SPVe), descending (spinal or inferior) vestibular nucleus; iMVST, ipsilateral medial vestibulospinal group; iR-VO, ipsilateral vestibulo-ocular group; Is, isthmus, LVe, lateral vestibular nucleus; LVST, lateral vestibulospinal group; r, rhombomere; SC, spinal cord; SVe, superior vestibular nucleus.

The relationship between hodologically defined vestibular groups with classically defined nuclei is summarized in Figure 6.

### Commissural Axons

Commissural axons associated with some of the groups also occupy specific rhombomere-related territories. Although the vestibulocerebellar neuron groups in the chicken collectively occupy large stretches of the vestibular column (except r4 and r5), their commissural fibers are more restricted, indicating a precise molecular control of axon navigation. Commissural axons from r1-r3 cR-VC neurons are restricted to r2, whereas commissural axons from r6-r7 cC-VC neurons cross the midline in r6-r8 together with auditory axons from the magnocellular nucleus to access the inferior cerebellar peduncle (Díaz and Puelles, 2003). The cMVST axons cross the midline in r5 (Díaz et al., 1998).

## How Conserved Is the Vestibular Efferent Hodological Mosaic?

Relating the hodological mosaic of the vestibular projection neuron populations to rhombomeric segmentation has only been performed in a few species of fish, amphibians, birds and mammals (Pétursdóttir, 1990; Glover and Petursdottir, 1991; Jansen, 1991; Suwa et al., 1996; Díaz et al., 1998, 2003; Straka et al., 2001, 2002; Díaz and Puelles, 2003; Pasqualetti et al., 2007; Di Bonito et al., 2015). Rhombomeres 1–8 all give rise to hodologically defined vestibular projection neurons, but small, presumably non-projection neurons have also been associated with the caudalmost rhombomeres r8-r11 (Lipovsek and Wingate, 2018). The vestibulocerebellar neuron populations have been studied only in chicken embryos and larval *Xenopus* (Straka et al., 2001; Díaz and Puelles, 2003), and the vestibulo-vestibular neurons only in frogs (Malinvaud et al., 2010). The vestibulo-thalamic and vestibulo-reticular projections have yet to be explored in any species in a rhombomeric context.

**FIGURE 7:**
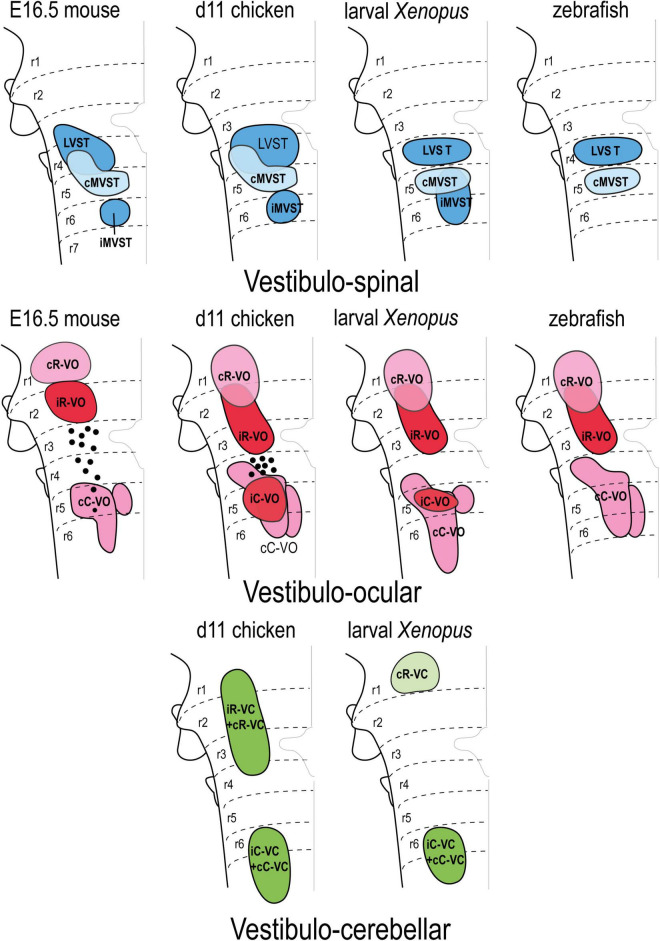
Correlation of the pattern of the vestibular projection neuron groups with rhombomeres in the indicated amniote and anamniote species [reported data from Pétursdóttir (1990); Glover and Petursdottir (1991); Jansen (1991); Suwa et al. (1996); Díaz et al. (1998, 2003); Straka et al. (2001, 2002); Díaz and Puelles (2003); Pasqualetti et al. (2007); Di Bonito et al. (2015)]. Modified from Díaz and Puelles (2019) with permission. cC-VC, contralateral caudal vestibulo-cerebellar group; cC-VO, contralateral caudal vestibulo-ocular group; cMVST, contralateral medial vestibulo-spinal group; cR-VC, contralateral rostral vestibulo-cerebellar group; cR-VO, contralateral rostral vestibulo-ocular group; iC-VC, ipsilateral caudal vestibulocerebellar group; iC-VO, ipsilateral caudal vestibulo-ocular group; iMVST, ipsilateral vestibulo-spinal group; iR-VC, ipsilateral rostral vestibulocerebellar group; iR-VO, ipsilateral rostral vestibulo-ocular group; LVST, lateral vestibulo-spinal group; r, rhombomere.

Based on available data, the hodological mosaic is highly conserved, with a number of elements found consistently in all species. However, variations do exist, as summarized in Figure 7. Particularly those neuron groups associated with r1-r3 and r4-r6 tend to share hodological features. The ipsi- and contralateral rostral vestibulo-ocular groups (iR-VO and cR-VO) and ipsi- and contralateral rostral vestibulo-cerebellar cell groups (iR-VC and cR-VC) lie rostral to the r3/r4 boundary in all species examined. In r4-r6, the vestibulospinal groups are mainly segregated (LVST at r4, cMVST at r5, and iMVST at r6) but there is limited overlap between the LVST and cMVST in r4, and between the caudal vestibulo-ocular and vestibulocerebellar cell groups (iC-VO, cC-VO, iC-VC, and cC-VC). In the chicken embryo, the degree of this overlap was analyzed and quantified in three-dimensional digital models, and was similar to that seen for MN pools in the spinal cord (Díaz et al., 2003). The pairs iR-VO and LVST, iC-VO and LVST, and iC-VO and IMVST were essentially non-overlapping, as they are arranged in a rostrocaudal sequence (i.e., iR-VO, LVST, iC-VO, and iMVST along r2-r6). By contrast, the cMVST and cC-VO groups were highly overlapping in r5 and a distinct population of dual-projecting neurons (thus belonging to both groups) was also present (Díaz et al., 2003).

Some of the groups are restricted to a single rhombomere, including the LVST and cMVST groups in frogs and fish [Suwa et al., 1996; Straka et al., 2001, 2002; LVST derives from a single rhombomere (r4) in mouse, although there is a r4-derived component in r3; Auclair et al., 1999; Di Bonito et al., 2015], the iMVST group in mouse and chicken (Díaz et al., 1998; Pasqualetti et al., 2007) the iC-VO group in chicken and Xenopus (Díaz et al., 1998; Straka et al., 2001, 2002), and the cR-VO group in the mouse (Pasqualetti et al., 2007). By contrast, other hodologically defined cell groups are plurisegmental (for example, the cC-VO group in all studied species).

Some notable interspecies variations include: in teleost fish, only two vestibulospinal and three vestibulo-ocular projection neuron populations have been identified (Suwa et al., 1996), the iC-VO group appears early but is not seen in the late mouse embryo (possibly due to axon retraction to supraspinal levels, Pasqualetti et al., 2007), and the iR-VO group has not been observed in larval *Xenopus* (Straka et al., 2001, 2002).

Interspecies variations have also been described in the mediolateral axis (Figure 7); indicating that comparative studies of the dorsoventral sources of these neuron groups are needed.

## Conclusion

During the last 30 years, a segmental interpretation of morphological, molecular, genetic, and developmental data has contributed to clarifying the anatomical and functional organization of the vestibular column. The rostrocaudal rhombomeric patterning, wherein each rhombomere has a unique gene expression signature, correlates with hodologically defined vestibular neuron groups with specific functional identities related to axon trajectory and synaptic target. The hodologically defined vestibular neuron groups are unisegmental or plurisegmental in origin; in contrast, the classical cytoarchitectonic nuclei are mainly plurisegmental. Moreover, the hodological neuron pattern is highly conserved through vertebrate evolution.

## Author Contributions

CD: conceptualization, writing, and figures. JCG: conceptualization and writing and revision. Both authors contributed to manuscript revision, read, and approved the submitted version.

## Conflict of Interest

The authors declare that the research was conducted in the absence of any commercial or financial relationships that could be construed as a potential conflict of interest.

## Publisher’s Note

All claims expressed in this article are solely those of the authors and do not necessarily represent those of their affiliated organizations, or those of the publisher, the editors and the reviewers. Any product that may be evaluated in this article, or claim that may be made by its manufacturer, is not guaranteed or endorsed by the publisher.

## Footnotes

1
https://developingmouse.brain-map.org/
